# P-158. Association Between Clostridioides difficile (C. diff) Test Positivity and Colorectal Cancer (CRC) in Adults in a Multisite Hospital-Based Retrospective Cohort Analysis

**DOI:** 10.1093/ofid/ofaf695.382

**Published:** 2026-01-11

**Authors:** Sean M Anderson, Samara Rifkin, Xingyu Chen, Cynthia L Sears, Matthew Robinson

**Affiliations:** Johns Hopkins Medicine, Baltimore, MD; University of Michigan Medicine, Ann Arbor, Michigan; Johns Hopkins Medicine, Baltimore, MD; Johns Hopkins University School of Medicine, Baltimore, Maryland; Johns Hopkins University School of Medicine, Baltimore, Maryland

## Abstract

**Background:**

Recent mouse model data revealed that chronic infection with certain strains of toxigenic *C. diff* induces colorectal neoplasia. However, epidemiologic links between *C. diff* infection and human CRC development are incompletely explored.

**Table 1:**
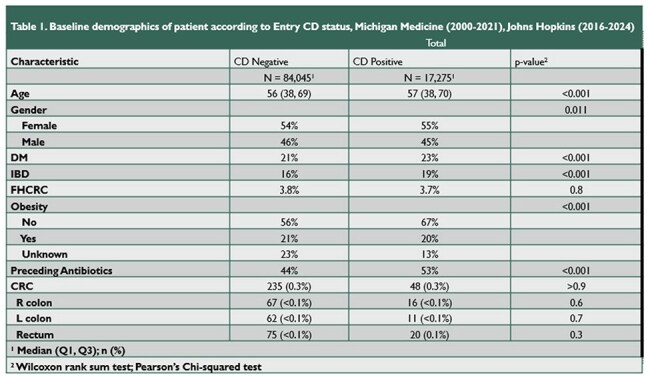
Baseline Demographics of Patients According to Entry CD Status

A comparison of baseline demographic, medical history, and outcome factors comparing the two primary cohorts of this study. Continuous variables were compared using the Wilcoxon rank sum test, categorical variables using Pearson's Chi-squared test.

**Table 2:**
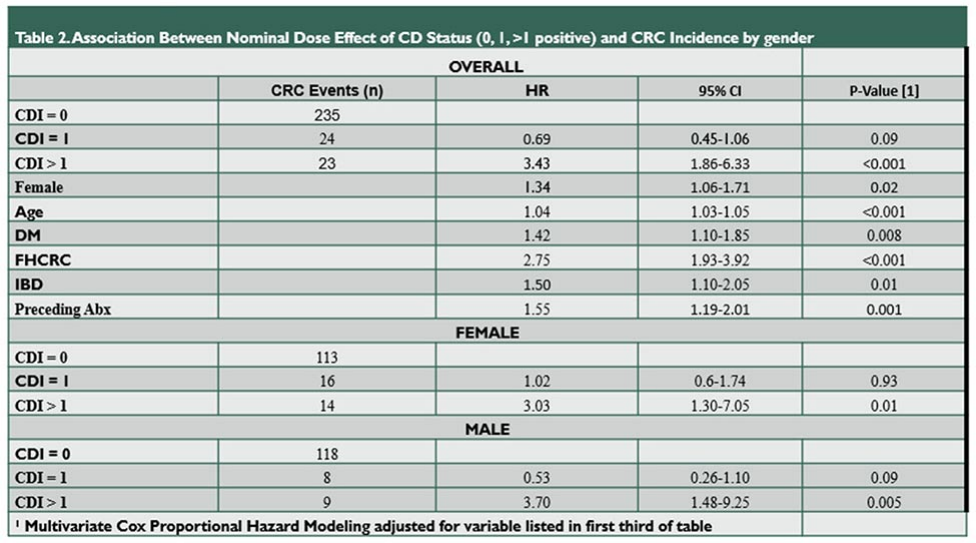
Association Between Nominal Dose Effect of CD Status and CRC Incidence Stratified by Gender

A comparison of the outcome of interest, incident CRC development >1 year after cohort entry, stratified by classification of C diff testing (negative, positive once, persistently positive) and by gender. The additional categories listed in the upper half of the table contain the variables used for adjustment in the final Cox proportional hazard model.

**Methods:**

We retrospectively analyzed a longitudinal dataset combined from two academic medical centers. All adults tested for *C. diff* by a stool-based test were stratified into *C. diff* positive and negative cohorts based on the results of their first test. Participants were also classified as persistently positive if at least one additional positive *C. diff* test > 30 days after entry was documented. Participants with CRC prior to testing or < 1 year from entry were excluded. Participants were censored at death, CRC diagnosis, or last encounter (minimum 1 year of follow-up required). At study entry, comorbidities and demographics were analyzed. The primary outcome of interest was new acquisition of CRC based on compatible ICD10 code. Multivariate Cox proportional hazard modeling adjusting for seven variables known to contribute to CRC was performed comparing incidence rates of CRC between the two cohorts.

**Results:**

101,320 individuals were included in this analysis (17% *C. diff* positive cohort). Those who tested positive were older (57 vs 56 years, p< 0.001), more female (55% vs 54%, p=0.01), and a higher percentage had inflammatory bowel disease diagnoses at entry (19% vs 16%, p < 0.001). There was no significant difference in Elixhauser Score (median 5.00 with IQR 0-12 in both cohorts) or history of polyp disease prior to enrollment. Our primary outcome occurred in 48 individuals positive for *C. diff* and 235 individuals negative for *C. diff* (both 0.3%, p >0.9). Multivariate analysis showed no significant difference in incident CRC development between the two cohorts (AHR 1.04 [95% CI 0.76-1.44]); however, sub-analysis of the multiply *C diff* positive cohort revealed an increased risk for incident CRC (AHR 3.43 [1.86-6.33, p< 0.001]).

**Conclusion:**

This retrospective analysis demonstrated a significantly increased risk for incident CRC in adults who persistently test positive for *C. diff*. Future analysis with larger, diverse cohorts could be useful to further evaluate the impact of *C. diff* infection on CRC.

**Disclosures:**

All Authors: No reported disclosures

